# Decentralizing Online Food Delivery Services: A Blockchain and IoT Model for Smart Cities

**DOI:** 10.1007/s11036-023-02119-5

**Published:** 2023-02-20

**Authors:** Ulpan Tokkozhina, Bruno Miguel Mataloto, Ana Lucia Martins, Joao C. Ferreira

**Affiliations:** 1 Instituto Universitário de Lisboa (ISCTE-IUL) Business Research Unit (BRU-IUL), 1649-026 Lisbon, Portugal; 2 Instituto Universitário de Lisboa (ISCTE-IUL), ISTAR, 1649-026 Lisbon, Portugal; 3 INOV Instituto de Engenharia de Sistemas e Computadores Inovação, 1000-029 Lisbon, Portugal

**Keywords:** Blockchain, Smart City, Food distribution process, Internet of Things (IoT), LoRa, Supply chain

## Abstract

With the worldwide pandemic outbreak, the style of restaurant food consumption underwent a major shift towards online delivery services. This poses an urgent need in trust between main stakeholders of the process: requiring restaurants to correspond to the quality declared, providing high quality and safe to consume meals, and obliging delivery entities to deliver food carefully, scrupulously following the delivery conditions. In this research, we explore a novelty approach that combines IoT, Blockchain technology, and city LoRa network to create a new trusted, decentralized approach for food distribution process in the context of a Smart City. This approach allows controlling the food delivery process using sensors data to control live location, temperature, vibrations, and shakings during the transportation process. We also suggest a fresh perspective on a rating system of delivery entities, where reputation points will be provided both from the side of the restaurant and the final consumer. This will create more trust towards the delivery entity since information will be tamper-proof and immutable due to the nature of Blockchain. This novel system proposal allows rethinking the online food delivery process in the context of Smart City, using the city’s LoRa LPWAN radio frequency technology and Blockchain decentralized solution.

## Introduction

Many food delivery platform providers (e.g., UberEats, Just Eat, GrubHub, DoorDash, Deliveroo, Boltfood) have entered the market in recent years with various business models in cities worldwide. It was anticipated by [1] that by 2025, the global food delivery market can be worth more than 20 billion dollars. These platforms are characterized by the fact that they do not run kitchens, instead they focus on marketing the meals of their partners, consolidating all order-related operations in simple smartphone apps, and handling delivery via a fleet of either staff or crowd-sourced subcontractors. As per [2], restaurants can get a new source of income with the help of food delivery platforms, without increasing the number of staff or seats. The same authors mention that consumers are also benefiting by having more choices and variety of meals, also having access to relevant information such as reviews about a chosen restaurant. Following the Covid-19 outbreak, the world is witnessing a huge shift in trends of how people buy and consume food. As noted by [3], unlike hospitality industries such as tourism, hotels, entertainment, etc., that faced a threat to the business and a radical decline, the online food delivery industry grew radically. Using food delivery platforms and apps became a routine, leading to various social campaigns, such as “Support SME” campaign in Thailand that was introduced by Food Panda delivery platform, which reduced the commission rates and fees for SMEs (small and medium enterprises) to support local restaurants and cafes [4]. Similar campaigns were launched in various countries, which helped some small local businesses go through this pandemic period. In the light of the Covid-19 outbreak, a study from the point of view of the final consumers [5] was conducted and revealed that factors such as price, information quality, and promotion have the biggest impact on overall customer satisfaction. The same research also refers that usability factors, such as navigational design and perceived ease of use are not playing a significant role to customer satisfaction and loyalty. Some criticism exists towards food delivery platforms [6] from both economic and social perspectives. Even though online food delivery platforms build new jobs and sales opportunities, the high commissions that it charges to restaurants and the working conditions for the delivery entity are still questionable. From a social perspective, food delivery platforms also tend to affect the relationship between final consumers and the food that they order - issues such as public health influence and traffic systems outcomes persist [6].

In a typical food delivery application, a user registers by his/her name, address, and location. After that, users are redirected into the app that shows nearby restaurants with delivery menus and offers, where upon choosing the desired restaurant, the user entrusts food to the truck/bike delivery service. The delivery entity collects the meal and uses the guide/map to deliver it to the user (customer), where the customer is also able to see the identity and contact information of the delivery entity. Thus, the current architecture of food delivery platforms and apps is highly centralized and is also being criticized from the perspective of the high commission fees that food delivery platforms charge to restaurants. The price of the food on the menu is required to be the same as it would be in a restaurant, but then extra fees are being charged from restaurants for these platforms to cover expenses such as being a delivery partner, for customer support, etc. According to [7], orders on Deliveroo are the most expensive among online food delivery companies, costing on average 31% more than ordering directly from a restaurant, UberEats costs extra 25%, and Just Eat orders cost 7% more.

Blockchain technology (BCT) can be characterized as a technology with inherent distributed and immutable features [8], which provides an infrastructure for the activities to take place in a transparent, secure, and traceable environment without the need of the central entity. Operations within BCT are entirely decentralized and do not rely on a central entity (third party) as all the transactions are automatically verified with smart contracts [9]. Smart contracts are conditioned by the ability to autonomously verify and execute the terms of a given prebuilt contract [10]. This distributed ledger system is considered for potential applications in various e-commerce products, including food products, health medicines, electronics, security appliances, etc. [11].

Therefore, the purpose of this study is to investigate the ability of a BCT-based solution in decentralizing the current architecture of food delivery. The proposed solution will combine IoT and a city LoRa network. Thus, the goal of this study is to explore the potential of the combination of BCT, IoT, and LoRa network in improving the food delivery system by creating a proposal for a collaborative environment with no centralized entity involved, where the food delivery process can be enhanced through sensors tracking.

This paper is built as follows: Sect. 2 is reviewing the current literature and research in the field, Sect. 3 introduces the methodology used for the study, Sect. 4 presents the proposed system, and is further divided into subsections of the proposed system constituent – blockchain, sensors, and others. Sect. 5 provides a discussion of the proposed system and Sect. 6 is concluding with contributions, limitations, and topics for future research.

## State of Art

The concept of BCT was disseminated in a peer-to-peer version of electronic money called Bitcoin [12]. As the technology supporting Bitcoin, blockchain has become a major research topic in the financial industry. The immutable nature of BCT [13] already showed success when applied to securely validate transactions in the financial industry, and it looks promising for use in terms of food quality monitoring in global supply chains (SCs) [14].

In [14] it is claim that BCT, when combined with other digital technologies, poses budding capabilities to various players in food SCs to share information in a secured manner, controlling processes, and allowing traceability of food that may prevent potential risks to the final consumer. However, there is still an observable lack of comprehensive and convincing business use cases on the adoption of BCT in the food SC area, thus uncertainties from the side of practitioners still exist due to technology knowledge scarcity, and it creates a potential barrier for adoption [15].

Authors [16] went through several possible smart detection strategies that can be applied to classical food SCs, for example portable detecting devices, IoT, cloud computing, among others. One of the solutions that was introduced by [16] is smart packaging, which can bring transparency through monitoring the environmental conditions of the packed food. Consequently, by using indicators and sensors in the food packaging itself, it will be possible to trace the information about the food origins and its condition history. However, when talking about the traceability feature that BCT brings, it is important to remember that, for food, what consumers care about in terms of traceability is not only the information about origins but also what happens through the process while the product moves across the chain [17]. Findings from [18] show that dimensions of food traceability systems, such as the quality of the information, reliability, and product diagnostics, have an impact on the perceived value for the consumer and positively affect the purchase intention.

Food tracking and quality control in delivery companies are essential to ensure that customers get the requested orders on time and in the best possible conditions. Food delivery companies or applications are currently built upon centralized platforms with a three-sided marketplace business model, which connects restaurants (that pay a commission to the platform) with final consumers (who pay small delivery fees) and drivers (who earn based on the timely and reliable deliveries): the food delivery platform is therefore responsible for management and payments [19]. The online food ordering system is beneficial for both (1) restaurants, because it creates new opportunities to increase sales and expand their business, and (2) customers, as it provides convenience in online food ordering [20].

Supply tracking platforms use GPS to track the driver’s real-time location [21–23] together with a customer support feature in case the customer has an issue with the order. These types of systems mostly disregard food transport quality, focusing on delivery efficiency. In many countries, couriers use GPS applications such as Google Maps or Waze [24–26] to travel from the pickup location to the customer address, however, sometimes the most efficient route can compromise food quality and temperature due to poor road conditions or driving performance.

In some food SC contexts, road conditions such as vibrations during transportation play an important role in quality and marketable condition wastage [27, 28]. Cold chain logistics (or temperature control logistics) for modern food SCs has been using RFID sensors over the logistic network to carry out the purpose of timely control of the storage and food temperature throughout the logistics process [29]. RFID sensors are usually installed on the outside package and can track temperature and humidity changes during transportation [30], [31]. A smart RFID tag developed in [32] collects sensor data during transportation, and results can be extracted after the product is delivered. The authors [33] modified a passive RFID tag with a copper-doped ionic liquid, which resulted in a possibility for simultaneous acquisition of data from multiple packages with the same reader without any need for visual or direct contact with it. Some of the limitations that affect the intelligent packaging adoption and implementation into business operations of SCs include the business type and size, available capital, integrated operations standards, among other issues [34].

A sensor network was developed by [35] to monitor air temperature inside transport vehicles, composed of temperature sensors and a base station inside the vehicle. The sensors collect data and send it to the base station, which stores the data for further analysis. The authors also implemented a warning mechanism for when critical temperatures are detected, which sends a warning message to a mobile phone. Existing cold chain systems can collect real-time data, though accessibility is limited to the beginning and the end of the journey. RFID tags can be a good solution for a more high-cost type of products, since they need to be individually placed on each package, but in terms of hot food delivery it would be a time-consuming approach.

Based on the fuzzy multi-criteria decision-making approach, [3] revealed that when evaluating online food delivery platforms, factors that are playing a major role for final customers (consumers) are the delivery speed, online service level, fulfilment of orders, convenience of payment, and the delivery cost. The authors [36] further confirm the influence of the food delivery platform service quality on customer loyalty, claiming that key factors to refrain loyalty of current customers is suitability and similarity between performed and delivered orders and displayed images on the application.

To apply novel technologies for food traceability systems, it is important to first make sure that it will contribute to enhancing trust in food safety from the perspective of a final consumer [37]. The same authors posit that, consequently, it is crucial to inform consumers how exactly the system works and to gain their understanding and confidence. In the current architecture of food delivery, customer orders from the application, where cost is divided between the restaurant, delivery entity, and the platform itself, courier payments reflect the amount of fulfilled orders, the time spent on deliveries, and general demand at the delivery time [38].

Building on the literature available, in our approach, we intend to create a decentralized environment, which will remove the need for a third-party, thus also resulting in cost efficiency. We build upon the idea of using a real-time sensor network that can be tracked remotely in a distributed decentralized manner and does not require individual sensor placement on each meal. Since sensor devices will be placed on the courier’s (delivery entity) backpack, we are suggesting a novel trustworthy view on the delivery process of food bought online.

## Methodology

This study is focused on an acute contemporary business phenomenon, with the purpose of investigating the ability of blockchain-based supply chain (SC) to decentralize and promote trust in food delivery services. To address the purpose of the study, the obstacles of the current centralized food delivery platforms were investigated through the prior literature. We assume that assuring a quality meal in a food delivery supply chain, and at the same time making the food preparation status and delivery conditions transparent in real-time, can potentially increase the trust of the final consumer towards the selected platform, thus also enhancing the overall experience and trust towards both the restaurant and the delivery entity.

In a multidisciplinary team of technical and managerial experts, we started by reviewing the current literature in the field, to identify the resistances and constraints in the industry. As a next step, we focused on various technologies, that could be integrated to decentralize online food delivery. Based on the chosen technological solutions, we developed a proposal to control the distribution process of food delivery using IoT sensors and LoRa network on an example of the Lisbon area.

To fulfill the goal of this study, we propose a decentralized BCT platform powered by IoT sensors and LoRa network. As food delivery became a more common service worldwide due to pandemic restrictions, the proposed system addresses the issue of quality and trust in food delivery platforms among different players.

## Proposed System

**Fig. 1:**
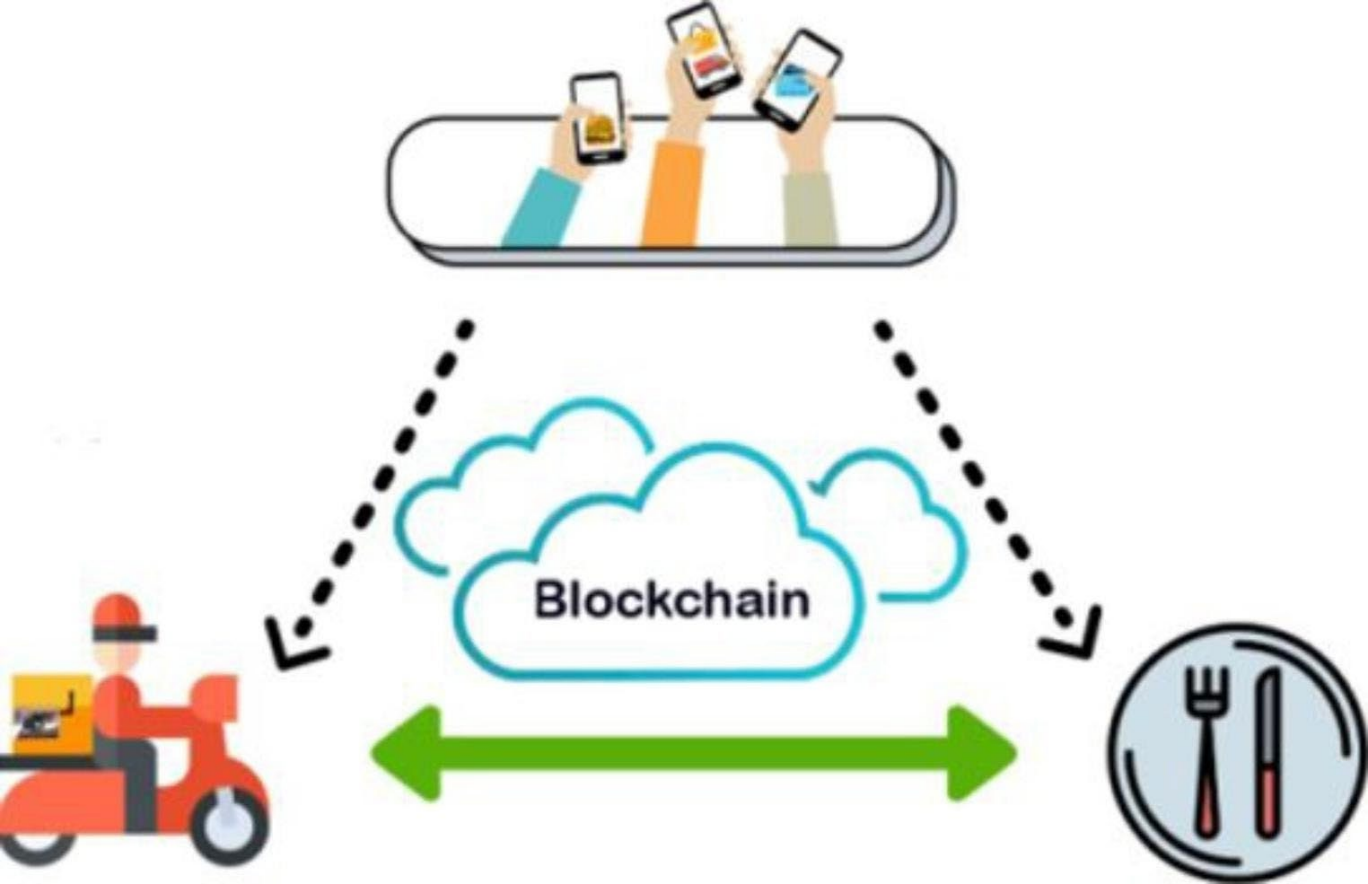
Three-sided delivery system overview

In this research, we propose a decentralized food delivery blockchain network (Fig. 1), where the centralized management platform is diluted on the three-sided parties: restaurants, delivery entities, and final consumers.

New wireless protocols, known as Low Power Wide Area Networks (LPWAN), are claiming to execute the long-range communication at the low power, by providing high coverage capabilities while allowing high energy efficiency [39]. Driver’s reputation ceases to come from delivery time and instead it is determined by the LPWAN of Internet of Things (IoT) sensors that monitor food transportation quality and characteristics such as temperature and vibration inside the transport container, also enabling users to review real-time location throughout the whole journey. Sensors monitoring the food transport conditions are not only allowing final consumers to gain trust, but they also provide a base for an accurate driver evaluation, letting restaurants choose drivers based on adequate criteria for the food to be transported.

Smart city technologies, such as IoT sensors and LoRa network, powered by BCT-based decentralized system provide a way of automating activities, at the same time minimizing costs, and supplying final consumers and all the system users with real-time services [40]. Decentralization of systems results in lower costs due to the multi-stakeholder tracking, which does not require a third-party verification (in our case a food delivery platform provider), because all records and transactions are immutable and verifiable [41]. BCT-based platforms are naturally incorporating data from all participating parties, continuously verifying it without any need for additional staff or time-consuming processes, which leads to reduced costs of the verification processes [42]. In our approach, cost-efficiency is achieved through the fact that commissions that were intended for the management platform itself are now distributed between all parties, thus reducing the delivery cost for the final consumer and the fee that restaurants are current paying to centralized platforms.

### Blockchain

Blockchain is a decentralized, unmanipulated distributed ledger, where data is stored in a block structure and verified by the consensus of the participants in the system. BCT uses distributed architecture, cryptography, consensus algorithm, smart contract, and other technologies to achieve tamper-proof, forgery-proof, and traceability information in the process of information collection, circulation, and sharing [43].

The use of blockchain technology brings some benefits such as anonymity [44], immutability [45], transparency [46], security [47], and fast transactions [48].

Anonymity is an important feature of this technology that attracts both individuals and organizations in various sectors of business to implement it [49]. Despite not being a key aspect of our proposed system, it is still essential to keep data restricted to a need-to-know policy between parties. Sensitive information about clients must not be available to drivers and restaurant contracts with drivers must not be available to customers. Blockchain allows users to be identified only by public keys, an essential element of the cryptosystem. This feature allows any person or organization to transact any sum of money anywhere in the world without government intervention and transaction costs are low [50]. Immutability is a fundamental feature of blockchain and has been identified as one of the reasons for its success to date [45], [51]. By its design, changing a blockchain would mean changing every following block, as each block contains information from the previous block [12]. This is infeasible at the linear rate at which the chain expands, with new blocks being issued approximately every ten minutes [52].

One of the main issues in current early state of BCT applications is the scalability problem. It is characterized by limiting the amount of data and transactions that can be processed and stored in a short amount of time [8]. As the shared ledger will grow fast, each participant needs the full ledger access to be a part of the network – experts propose addressing this challenge through vertical optimization including new tools, and expect that solutions for specific use cases will evolve. [50]

BCT can support identity management of persons and objects that is fundamental in collaborative processes to know who performed a certain action. In our proposal, it is important to highlight the collaboration and identification transparency, as this will create value for the system itself – restaurants and delivery entities are going to receive reputation points based on the quality of their services. The chain blocks allow us to know who did what and how, and IoT sensors also register these activities (e.g., time, temperature and humidity conditions, vibration/shaking process). Smart contracts allow registering and defining transactions among stakeholders based on pre-defined conditions, thus automating the process. One of the important predefined conditions should be an individual access to the nodes, with a proper authorization based on the identity of a player. All stakeholders need to join by the web application or mobile device creating an initial identity and associated profile. It means, that for example, final customer would not have the same authorization on the network, as a restaurant – such architecture would secure the network from potential attacks and information altering.

In the current industry of food delivery, central entity organizes the environment, creates and settles the app and then invites restaurants and delivery entities to participate by winning a commission. In BCT application, the difficulty might arise from the very beginning of “Where to start and who should run this?”. As BCT does not have a central entity [9], it means that initially there should be a group of confederates (e.g. a group of restaurants), who would make an effort in agreeing on the implementation architecture. As a result, BCT-based solution would be built, and then it will be up to raising awareness among the industry, to gain recognition of benefits caused by joining a decentralized network. Therefore, this study provides a comprehensive plan for the confederates’ implementation.

### Monitorization System Based on IoT Sensing

Internet of Things systems are becoming a reality for smart cities all around the world. As per [53], IoT is a key infrastructure for information integration and transmission in a context of smart city implementations that aims to obtain effective city management, efficient operations, and improved experiences, and higher life quality of city residents. LPWAN are low power sensor networks that provide large coverage with minimal energy consumption, which emerges as the best option for an IoT application such as our food monitoring system. LoRa is a LPWAN radio frequency technology (868 MHz in Europe) already deployed on many cities around the world [54–56], with free or paid solutions. LoRa allows cities to be covered with a small number of base stations and does not require a gradual rollout when compared to traditional networking [54]. Being a radio frequency, LoRa gateways transfer radio packets to the Internet and deploy them to an endpoint such as an end-user application or a database. In the implementation referred to in this paper, we focus on the city of Lisbon, in Portugal, where both solutions are already available [57]. Consequently, it is only necessary to develop the sensors to be used by the delivery entity.

### City Coverage

**Fig. 2:**
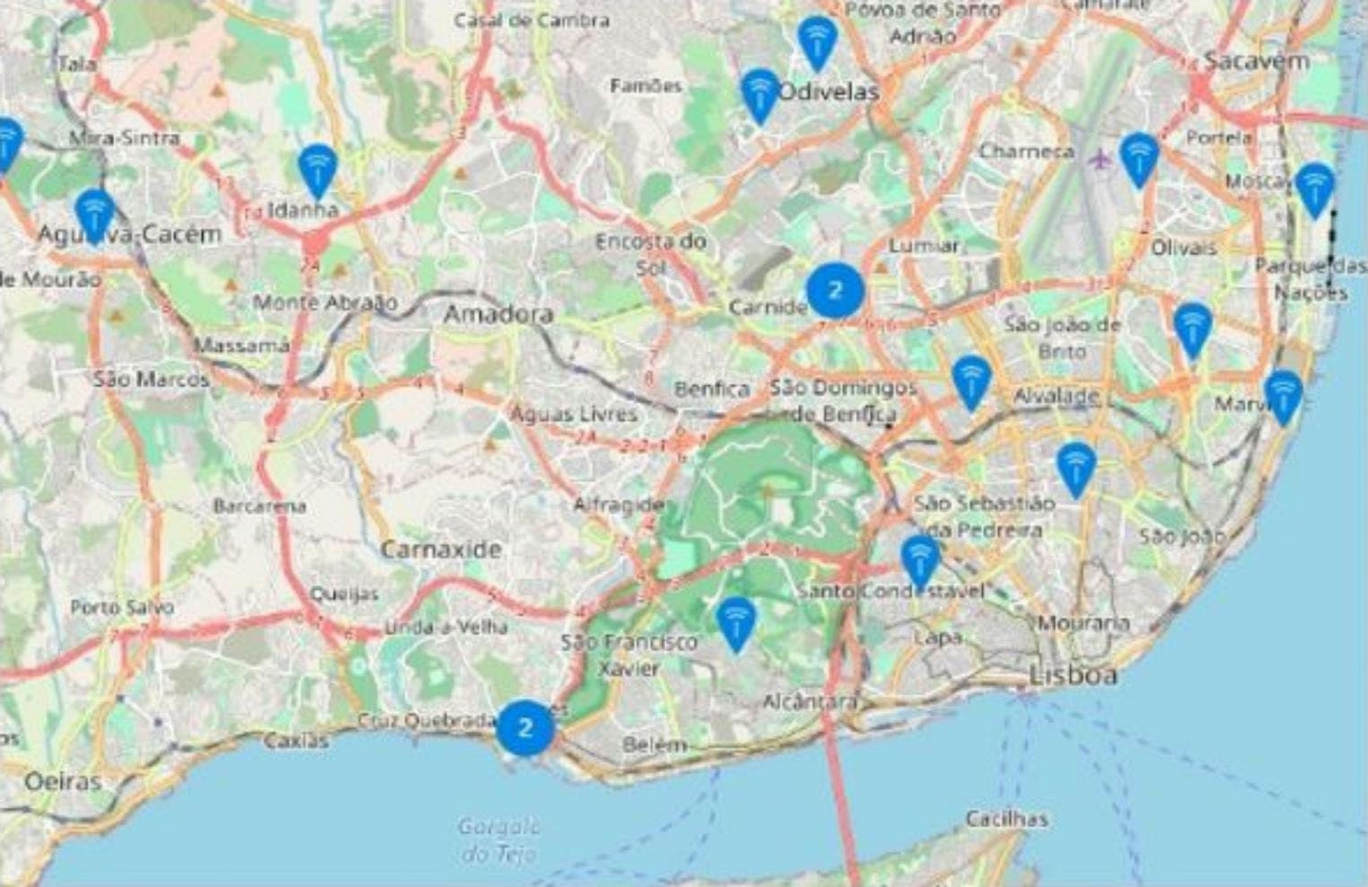
Coverage map of The Things Network in Lisbon

TTN, or The Things Network, is a worldwide free-of-use LoRa network, with 21,952 gateways or access points deployed in 151 different countries at the time this paper was written, where data transmitted by a node is usually obtained by multiple gateways [58]. There are currently 145 TTN hotspots in Portugal, 26 of them located in the Lisbon Metropolitan Area. With an average range of approximately 18.5 km in rural areas and 6.5 km in urban areas [59], and with the current distribution in the city (Fig. 2), the network foundations are in place to develop a food tracking sensors’ network.

### Sensors

**Fig. 3:**
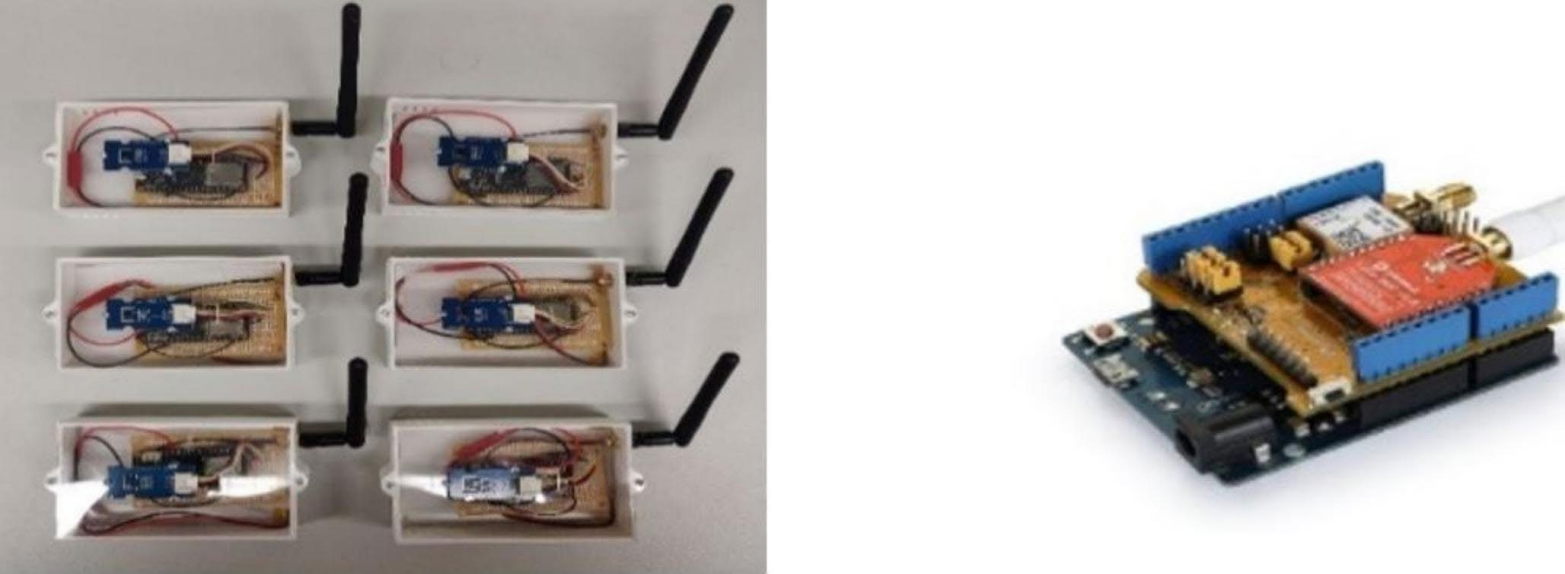
(a) (on the left) Sensor Devices prototype example with LoRa connectivity; (b) (on the right) Arduino with LoRa / GPS module

Sensor devices powered with a rechargeable battery (Fig. 3a), collect periodic data inside the courier’s backpack, transmitting over the LoRa network to the nearest gateway by radiofrequency.

These developed sensor devices consist of a LoRa enabled microcontrollers, such as lora32u4 used on sensor device prototypes from Fig. 3a, or Arduinos with a LoRa/ GPS module attached (Fig. 3b). Development microcontrollers can be equipped with any type of sensor, according to the application.

In this case, the following sensors were be assembled:

- GPS module to track the real-time location of a delivery entity;
- Temperature and humidity sensor (DHT22 sensor, with a temperature range from − 40˚ to + 50˚C and a precision error of 2–5%);
- Vibration sensor (SW-420 vibration sensor) to track the vibrations throughout the road, which can influence the quality of a meal.

Equipped with these sensors, the sensor device will perform an initial data processing and will send data periodically. This way it will prevent unnecessary data transmissions, e.g., when the temperature does not change or the driver stops, saving battery and reducing network traffic.

### Supply Blockchain

To implement our solution, we use the Ethereum technology. Ethereum is a decentralized platform capable of running smart contracts and decentralized applications using blockchain technology. An Ethereum-based solution was previously used in a similar IoT sensors equipped case, but for the global shipment of containers [60]. Digital, smart contracts will allow a decentralized consensus contingent term that benefit from a tamper-proof and self-execution automated mode [61].

**Fig. 4:**
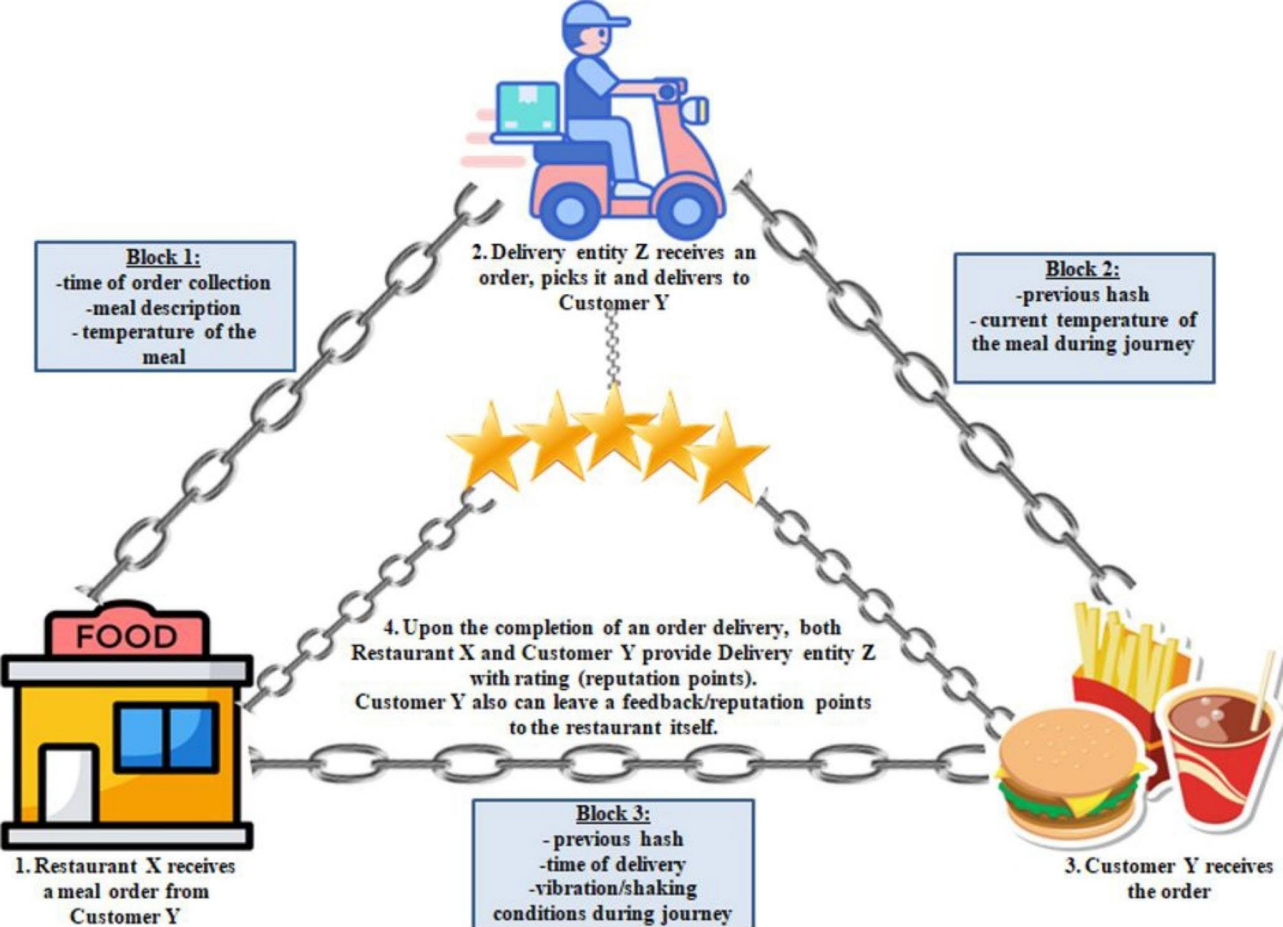
Blockchain-based system architecture in Ethereum

For the development of smart contracts, we propose using the Solidity language. This high-level language was created to allow the implementation of smart contracts that guarantee and govern the behavior of our BCT network. In [60] it showed efficient governance and management of a sender-receiver interaction. Figure 4 illustrates the architecture of our proposed system.

As previously stated, the main entities in this process are: (1) restaurant, (2) delivery entity, (3) final consumer. The smart contracts’ process starts with a consumer ordering a meal from a restaurant. Then the restaurant starts preparing the meal and the delivery entity sells a service, where the price is based on a transportation quality exploring: time, temperature conditions, distance, quantity, and vibrations on the road, or shaking conditions. All these features are predefined in the smart contract, which makes the process automatic and allows the city transportation (in this case a food delivery service) to operate without a central entity to manage (e.g., current central entities are the applications like UberEats, Just Eat, Bolt, etc.).

As our sensors send periodic data to the blockchain, the system only registers variables every 5 min: the maximum, minimum and average temperature and vibration values during that period. The GPS positions in the last 5 min are also attached to the message payload, allowing the system to trace the driver’s position.

## Discussion

Our system establishes a direct connection between the IoT devices and our blockchain solution, where each key entity stores data. Starting from the restaurant – delivery entity (courier) perspective, these have access to a list of registered couriers certified by the BCT. These delivery entities, or couriers, may already have some attributes defined or still unidentified, such as the driving performance and the quality performance, and the appearance of the meal delivered. The novelty of this decentralized approach is that not only all players can get access to real-time information at every stage of the process, but also the ability of the restaurants to choose a specific courier to deliver the order (the selection criteria can be based on previous reputation points gained by this specific delivery courier). By selecting a courier, a new transaction is initiated at the Blockchain. During delivery, the restaurant has access to real-time delivery information collected by the onboard sensors, including real-time location, food storage conditions, such as temperature and humidity, and noticeable vibrations (shakes) detected along the journey. After the delivery is completed, the restaurant can confirm or identify the courier’s attributes, based on the features tracked and give it some reputation points according to the delivery requirements. These couriers will benefit from a grading system, which may become the motivation for better performance and will lead to a higher number of future delivery requests from restaurants using the platform.

From the final consumer - courier perspective, after placing the order, the request is stored at the blockchain first as a pending order waiting for the restaurant confirmation. As soon as the order is picked by the courier, the status changes, allowing the final consumers to gain access to the same transport metrics as the restaurant: real-time courier location, food storage environment conditions (temperature and humidity), road vibrations, and any possible unexpected delivery events. After the meal is delivered, the final consumer will also have a possibility to confirm the courier’s identity, and give the rating points, or leave feedback on satisfaction with the services according to the delivery requirements, as well as providing the restaurant with rating points for the quality of the meal. This information will be stored in an immutable and tamper-proof manner, which will benefit not only in extra motivation for the high-level performance of the courier, but also in providing trust for other clients, who can always review the feedback.

One of the main novelties of this proposal is the immutability of information stored in the system and the trust built through transparency of information and reputation points. Final consumers gain full real-time visibility of progress information starting from the moment that restaurant is preparing food and all the way throughout the journey of the courier. Along with the driver’s delivery, the IoT device collects sensor data and updates it every 5 min (variables out of boundaries represent an anomaly along the way) together with GPS info. As the information on the BCT-based system is immutable and irreversible, the data can be later reviewed by any of the described players, and the reputation points can be given to both restaurant and courier based on the meal quality, delivery quality, appearance of the meal, etc.

The benefits of this application are that blockchain stores all orders regardless of their state, so the information that enters a BCT platform is immutable and cannot be altered. Given that all the main actors have access to the same veridical information about the delivery, being as well protected by a smart contract, transparency is achieved through the use of BCT, which allows building an extra layer of trust within all three stakeholders: restaurant – courier – final consumer. This decentralized approach is also specifically beneficial to the delivery entities. Couriers can gain transportation reputation on the decentralized platform, which comes from both restaurants and final consumers. It will not be based only on the number of deliveries performed and reliability, but on the storage and transportation conditions, which are crucial for the quality of the meal to both restaurants and final consumers.

## Conclusion

The presented decentralized application allows a new context of online food delivery systems for smart city implementations. The goal of this study was to explore the potential of the combination of BCT, IoT, and LoRa network for a new sensors tracking-based system proposal to be used for online food delivery improvement. This proposal showed a potential to build a trusted, collaborative environment with no centralized entity in charge of the process, where the food delivery process can be fully transparent for participants and further store important reputation information of restaurants and delivery entities. The difficulty can potentially arise in the implementation of BCT itself, as the central entity is not going to organize the process. Hence, this study also serves as a comprehensive plan of how the technology can be rolled out to the industry effectively.

The novel view on evaluation parameters is introduced in our system proposal, including temperature and humidity level trackers and a road vibration sensor, which allow both restaurants and final consumers to track possible unusual/undesirable actions throughout the delivery journey. Restaurants need to track the delivery parameters, as this influences the temperature of a meal by the time of delivery and the undesired vibrations on the road can negatively impact its appearance, which must be similar to what is represented on the menu’s image. From the view of a final consumer, it is also important to track the parameters, as they compose the overall trust towards the restaurant, the courier and influence the meal/service overall satisfaction.

The current rating of a delivery entity on centralized platforms is usually based on the number of deliveries performed by the entity and the satisfaction from the final consumer side. In our decentralized approach, it will also be possible to get the feedback and the rate to the courier from the side of the restaurant, which will be beneficial for other restaurants when choosing a delivery entity. Evaluation parameters in our case represent what is important for both final consumers and restaurants themselves: the real-time location tracking, temperature and humidity conditions, and the vibrations throughout the meal delivery. Compared to centralized systems, where the platform itself collects the fees, our approach eliminates the central entity and saves costs for all parties.

The use of LPWAN in our approach brings benefits in terms of high coverage capabilities and low power consumption, which may also be an important satisfaction factor for conscious and sustainable consumers. The type of evaluation parameters can also be improved in the future, and one may introduce new parameters for sensors to track. This approach is very flexible for application in other cities, as the entire system will work in any location where LoRa network gateways exist. As an alternative option for those cities where LoRa is not available, other LPWAN networks like NBIoT can be used. As this approach was based on the Lisbon city application example, we suggest applying this in other locations to embed the smart city for the food delivery systems.

This research proposes a system prototype of a BCT implementation to the food delivery industry, hence it was not tested in a real use case. We suggest for innovative industry representatives to take the initiative and use the proposed system and try to build a BCT-based environment for the food delivery process. The contribution of this study is limited by a specific application for online food delivery systems. As a suggestion for future research, we highlight the importance of exploring the usage of LPWAN networks and decentralized BCT solutions to track in other industries, where trust, storage conditions, and extra transparency are crucial: cold chain of raw meat, medication, dairy products, frozen semi-manufactured goods, and other perishable products.
